# Psychometric Properties of the Treatment Satisfaction with Medicines Questionnaire (SATMED-Q) in Patients with Diabetes, Arterial Hypertension and Heart Failure

**DOI:** 10.3390/ijerph19031088

**Published:** 2022-01-19

**Authors:** Natalia Świątoniowska-Lonc, Aleksandra Kołtuniuk, Beata Jankowska-Polańska

**Affiliations:** 1 Division of Internal Medicine Nursing, Faculty of Health Science, Wroclaw Medical University, 51-618 Wroclaw, Poland; aleksandra.koltuniuk@umw.edu.pl; 2 Center for Research and Innovation, 4th Military Teaching Hospital, 50-981 Wroclaw, Poland; bianko@poczta.onet.pl

**Keywords:** assessment tool, adherence, treatment satisfaction, psychometric

## Abstract

Background: Satisfaction with medicines is crucial in indicating patient experiences with the treatment which impact medicine adherence and compliance. Aim: The aim of this research was to adopt a general measure of patients’ satisfaction with medicines, the Treatment Satisfaction with Medicines Questionnaire (SATMED-Q), to the Polish language (BMQ-PL). Materials and Methods: A total of 197 patients qualified for the research, with the following diagnoses: type 2 diabetes (*n* = 42), hypertension (*n* = 55) and heart failure (*n* = 100), aged 64.73 (SD = 13.27). The criterion-related validity was determined with the use of a Polish version of the Adherence to Refills and Medications Scale. Confirmatory and exploratory factor analyses were used. Results: The respondents’ mean score was 73.63 (SD = 18.42). Cronbach’s alpha for the entire instrument was 0.847. All items of the questionnaire were found to have a positive item–total correlation. A multifactorial linear regression model showed that a significant (*p* ˂ 0.05) independent variable increasing the SATMED-Q score for the whole group was educational level (vocational education R = 14.576; secondary education R = 14.055; higher education R = 19.372) and low adherence (R = −1.384) was a decreasing variable. Conclusions: The present findings indicate a high level of reliability and validity of the translated SATMED-Q questionnaire, fully comparable to that of the original. The questionnaire can be used for the assessment of satisfaction with medicines among Polish patients.

## 1. Background

In recent years, there has been a strong interest among researchers in patient satisfaction, in a broad sense, as one of the determinants/results of care provided [1,2]. According to Shikiar [1], to consider patient satisfaction related to medical care, its three components should be distinguished: satisfaction with the health delivery system (includes issues of accessibility, patient–physician interaction, perceived quality of staff and facilities), treatment satisfaction (may include other procedures and therapies, e.g., activity limitations, dietary restrictions) and satisfaction with medication (impact on symptoms, side effects). However, it should be kept in mind that, despite the separation of these elements and the possibility of assessing them individually, they are strongly interrelated and interdependent.

Satisfaction with specific medications prescribed by physicians is defined as “patient’s evaluation of the process of taking the medicine and the outcomes associated with the medicine”. The assessment of satisfaction with medicines provides information on, inter alia, the ease/difficulty of taking the medicine, the occurrence of side effects associated with taking the medicine and impact of the medicine on daily life. This information allows physicians to modify the treatment plan and select the optimal medicine, taking into account the patient’s preferences regarding, e.g., medicine form or dosage [1].

Background satisfaction with medicines is crucial in indicating patient experiences with the treatment, which impacts medicine adherence and compliance. It has been shown that patients treated for chronic diseases, such as hypertension [3,4,5], diabetes [4,6,7], COPD [4,7], depression [4], epilepsy [8] or migraine [4], who are satisfied with medicines, are more compliant with treatment recommendations than those who are less satisfied. Oncology patients who are satisfied with their treatment also belong to the group of compliant patients [9,10].Additionally, it is well known that compliance with therapeutic recommendations determines the appropriate process of treatment, limiting the number of complications resulting from the progression of disease, and reducing the need for rehospitalization or hospitalization due to exacerbations.

The assessment of medication satisfaction is an important differentiating factor for chronic patients and should be continuously monitored as it affects patient outcomes in clinical practice [11,12]. Understanding factors that modify the medication satisfaction level will also be useful in improving clinical outcomes [11]. In order to assess satisfaction with medication, Atkins et al. [13] developed the Treatment Satisfaction Questionnaire for Medication (TSQM) in 2004 and in 2008. Ruiz [4] validated the Treatment Satisfaction with Medicines Questionnaire (SATMED-Q). Nevertheless, none of the questionnaires has been translated into Polish so far and no assessment of the psychometric properties of the tools has been carried out; therefore, the aim of this research was to adapt the original SATMED-Q tool to the Polish language (SATMED-Q-PL).

## 2. Material and Methods

During the study period, November 2019–February 2020, a total of 267 patients were selected. Of these, 39 patients did not meet the criteria to be included in the study or refused to participate. In the first stage of qualification, 228 persons who met the criteria were included, but 31 resigned, despite prior consent for participation. In sum, 197 people participated in the study, with diagnoses of type 2 diabetes (*n* = 42), hypertension (*n* = 55) and heart failure (*n* = 100), aged 64.73 (SD = 13.27) years, and who had been treated in a primary care clinic. Qualification for the research was carried out by a trained team consisting of two specialist nurses. All qualified patients rated treatment satisfaction after their most recent clinic appointment. Sociodemographic data were obtained from the medical register. The inclusion criteria were the following: written informed consent to participate in the research, age over 18 years, use of pharmacotherapy—taking minimum 1 medication for minimum 6 months due to hypertension, type 2 diabetes or heart failure—cognitive status allowing understanding of the purpose and methods of the research, and completion of the questionnaire (Mini-Mental State Examination: ≥18).

The research used two standardised questionnaires, as follows:

(1) The Treatment Satisfaction with Medicines Questionnaire (SATMED-Q)—a brief, multidimensional generic questionnaire (17 Likert-type items). The instrument is composed of six domains, exploring actual satisfaction with drug efficacy, side effects, convenience of use, medical care, impact on activities of daily living and general satisfaction. It also provides a total score for treatment satisfaction with medicines by adding up all domains. Totalling the direct scores of the items yields a total composite score ranging between 0 and 68. The resultant total composite score can be transformed to a more intuitive and easier to understand metric with a minimum of 0 and a maximum of 100 [4]. Permission to use the questionnaire and language adaptation was obtained from the Mapi Research Trust.
(2) The Adherence to Refills and Medication Scale (ARMS), which evaluates the patient’s adherence level. It comprises 12 items related to various aspects of non-adherence, scored on the following scale: 1—never; 2—rarely; 3—often; 4—most of the time. Therefore, total scores range between 12 and 48 points, with higher scores indicating poorer adherence [14,15].

### 2.1. Ethical Considerations

The study was approved by the local Bioethics Committee (approval no KB 42/2019). All participants gave written informed consent after thorough explanation of the procedures involved. All patients received information about the purpose and nature of the research and provided written informed consent to participate. All patients were informed of the purpose and nature of our research and provided their written informed consent to be included in it. All patients completed all questionnaires. The study was carried out in accordance with the tenets of the Declaration of Helsinki.

### 2.2. Translation Procedures

According to the adopted scheme, the questionnaire was pre-translated into Polish by two independent bilinguals with very good knowledge of both languages [16,17], in order to better reflect the nuances of the target language [18]. Discrepancies between the two translations were discussed by the team with the involvement of a third impartial bilingual translator who was not involved in the previous translations. Back-translation from Polish into the original language was then carried out to ensure the accuracy of translation [16]. The back-translation was carried out by a separate team of two people who knew Polish, but their first language was English. The research team, comprising the authors of the study and the author of the original version of the questionnaire, then reviewed all versions of the questionnaire to determine whether the translated and original versions achieved semantic, idiomatic, experiential and conceptual equivalence [16,17].

In order to test the initial version of the questionnaire, 30 patients of the cardiology department were qualified for the pilot study [19]. After completing the translated questionnaire, respondents were asked (either orally by the interviewer or by means of an open-ended question) to explain what they thought each questionnaire item and the corresponding answer meant.

### 2.3. Statistical Analysis

Analysis of quantitative variables (i.e., expressed as numbers) was performed by calculating the mean, standard deviation, median and quartiles. The analysis of qualitative variables (i.e., not expressed by number) was performed by calculating the number and percentage of occurrences of each value. Comparisons of qualitative variables across groups were made using the chi-square test (with Yates’s correction for 2 × 2 tables) or Fisher’s exact test where low expected numbers appeared in the tables. Analysis of the effect of multiple variables on the quantitative variable was performed using linear regression. The results are presented in the form of regression model parameters with a95% confidence interval. The quantitative variables were compared between two groups using the Mann–Whitney U test. The quantitative variables were compared between three groups using the Kruskal–Wallis test. When statistically significant differences were detected, the post hoc analysis was performed using the Dunn’s test to identify statistically significantly distinct groups. Correlations between quantitative variables were analysed using the Spearman’s correlation coefficient. The materiality level was assumed at 0.05. Therefore, all *p*-values below 0.05 were interpreted as indicating significant relationships.

The analysis was performed in the R programme, version 4.1.0 [20].

## 3. Results

### 3.1. Study Participants

The group with heart failure (HF) was significantly older than patients with hypertension (HTN) and diabetes (DM) (respectively: 67.59 vs. 62.51 vs. 60.83) and suffered the most comorbidities, took the most pills, and were those most frequently hospitalized for disease exacerbations, compared with patients with hypertension and diabetes (61.00% vs. 14.55% vs. 40.48%) (Table 1). The percentage of female respondents was highest in the group with type 2 diabetes and lowest in the group with heart failure (69.05% vs. 32.00%). The hypertension group was more diverse in terms of education than other groups—the hypertension group had both the highest proportion of people with primary education and higher education.

### 3.2. SATMED-Q Results

SATMED-Q general score, “Convenience of use” subscale scores (81.06 ± 25.18 vs. 73.33 ± 17.85) and “Impact on daily activities” (72.27 ± 30.26 vs. 62.17 ± 25.14) were significantly higher in the HTN group than in the HF group (Table 2). The “No side effects” subscale score was significantly higher in the HF group than in the DM group (90.33 ± 25.12 vs. 74.6 ± 33.08). The “Treatment Effectiveness” subscale score was significantly higher in the HTN and DM groups than in the HF group (70.76 ± 32.94 vs. 74.8 ± 28 vs. 63.08 ± 20.56). There were no differences in the domains among the groups compared—“Medical care” and “Overall satisfaction”

#### Analysis of Individual Items

A high floor effect was observed in questions 1–3 regarding the absence of treatment side effects (respectively: 4.1–4.6%; 76.1–77.2%).

### 3.3. Loadings

The loadings of each item ranged from 0.76 to 0.986 and were statistically significant (*p* < 0.05) (Table 3), meaning that all items correlated significantly with their subscale score.

### 3.4. Cronbach’s Alpha

In the Polish version of the SATMED-Q, the value of the Cronbach’s alpha coefficient was 0.847, which indicates very good psychometric properties and high internal consistency of the tool [21]. In the procedure for evaluating the psychometric properties of the scale, it is not necessary to exclude any of the items, due to their lack of influence on the Cronbach’s alpha values (Table 3).

### 3.5. Internal Consistency—Confirmatory Factor Analysis

In order to test whether the predetermined division into subscales fits the data, the confirmatory factor analysis (CEA) was performed. Because the SATMED-Q items are expressed on an ordinal rather than continuous scale, the diagonally weighted least squares method was used.

The original structure of SATMED-Q is a 6-factor structure. Satisfactory RMSEA, CFI, TLI and SRMR fit indices were obtained for this structure (Table 4).

### 3.6. What Is the Independent Variable of SATMED-Q?

Multiple factor linear regression model showed that a significant (*p* ˂ 0.05) independent variable increasing SATMED-Q score and thus increasing treatment satisfaction among type 2 diabetes patients was age (R = 0.633; 95%CI:0.105,1.161) and a variable decreasing the score was low adherence (R = −1.879; 95%CI: −3.45, −0.308) (Table 5). The R^2^ for this model was 59.15%, which means that 59.15% of SATMED-Q result variation was explained by the variables included in the model. The remaining 40.85% depended on the variables not included in the model as well as random factors.

Among heart failure patients, significant (*p* ˂ 0.05) independent variables that decreased treatment satisfaction were living alone (R = −8.71; 95%CI: −16.975, −0.445) and low adherence (R = −0.942; 95%CI: −1.635, −0.249). The R^2^ for this model was 20.34%, which means that 20.34% of SATMED-Q result variation was explained by the variables included in the model. The remaining 79.66% depend on the variables not included in the model as well as random factors.

For the entire group of subjects, an independent variable increasing treatment satisfaction was level of education (vocational education R = 14.576; 95%CI: 2.63, 26.522; secondary education R = 14.055; 95%CI: 2.096, 26.013; higher education R = 19.372; 95%CI: 7.167, 31.577) and the variable decreasing treatment satisfaction was low adherence (R = −1.384; 95%CI: −1.888, −0.88). The R^2^ for this model was 29.97%, which means that 29.97% of SATMED-Q result variation was explained by the variables included in the model. The remaining 70.03% depend on the variables not included in the model as well as random factors.

Among arterial hypertension patients, low treatment adherence had a negative effect on treatment satisfaction (R = −2.088; 95%CI: −3.402, −0.773).

## 4. Discussion

The objective of this study was to develop and examine the psychometric properties of a general tool for measuring treatment satisfaction that could be used in clinical practice for any chronic disease and any medication. This study is the first in which the SATMED-Q was translated into Polish and validated, with the questionnaire being translated from the original English version. The SATMED-Q is a reliable and valid tool for measuring treatment satisfaction. While performing translation and cultural adaptation, the team working on the Polish version of the SATMED-Q did not encounter any problems related to the adaptation of individual questions of the questionnaire.

The results of the study show that the SATMED-Q questionnaire is relevant, reliable, and can be used in everyday clinical practice, both as a unidimensional tool (using overall treatment satisfaction) and to measure patient satisfaction with different aspects of treatment (for which the tool’s subscales also proved to be relevant and reliable). Since few questionnaires have these characteristics, this feature makes the tool even more useful. When it comes to practicability, the response rate is highly satisfactory (almost all patients answered all questions) and the time to complete the questionnaire is very short, making it easy to use at any level of health care, especially in primary care where the time available to deal with patients is usually limited.

The Polish version of the SATMED-Q showed good psychometric properties and no cultural/linguistic differences were observed between the Polish version and the original version of the questionnaire. A high Cronbach’s alpha score (0.847) obtained in this study indicates good psychometric properties of the Polish version of the SATMED questionnaire. According to the literature, values above 0.7 are assumed to indicate good reliability of the scale [21]. In the study by Ruiz et al. [4] in a sample of 455 patients with chronic diseases, including type 2 diabetes, arterial hypertension, osteoarthritis, benign prostatic hyperplasia, chronic obstructive pulmonary disease/osteoarthritis chronic obstructive pulmonary disease/asthma, depression, and migraine, the SATMED-Q also showed high consistency and acceptable reliability.

The SATMED-Q seems to be a very well-prepared tool for assessing treatment satisfaction in patients with chronic diseases: original version α = 0.879 [4]; French version α > 0.87 [7]; validation study conducted among Spanish hypertension patients α = 0.916 [3]. Examination of responses based on the confirmatory factor analysis confirmed the originally proposed theoretical framework. The original structure of SATMED-Q is a 6-factor structure. Satisfactory RMSEA, CFI, TLI and SRMR fit indices were obtained for this structure. The observed relationship between different dimensions suggests that the scores from different subscales can be combined to form a meaningful total score.

The assessment of treatment satisfaction helps determine the benefits and convenience of taking the administered medications and is associated with increased adherence and greater willingness of the patient to continue taking the medication, can help predict the degree of patient compliance with treatment recommendations and improve the effectiveness of the administered therapy [22,23]. In this study, in the multiple factor analysis, the level of adherence to treatment recommendations was one of the factors affecting satisfaction with treatment both for the entire study group and for groups differing in terms of disease entities. The relationship between satisfaction and adherence is well established. Greater satisfaction is associated with better adherence, or, conversely, greater dissatisfaction is associated with lower adherence. This relationship has been demonstrated for a broad spectrum of diseases (e.g., rheumatic diseases, diabetes, hypertension) and in different settings (clinical and observational studies) [24]. In the study by Berhe et al. [25] conducted on hypertension patients being more satisfied increased the odds of being adherent. Among patients with heart failure, a lower score in the convenience domain compared with other dimensions is also reported and might be attributed to pill burden that poses difficulty to adhere to dose regiment [26]. Similarly, in the study by Horii et al. [27], involving diabetes patients, low treatment satisfaction was associated with low treatment adherence, suggesting that improving treatment satisfaction may improve adherence in patients with T2DM.

In this study, living alone appeared to be an independent variable of treatment satisfaction among patients with heart failure. People who live alone often complain about loneliness, which is the strongest independent variable determining dissatisfaction with doctors, health care, and Medicare Supplement plans [28], whereas old age was a variable affecting treatment satisfaction in patients with type 2 diabetes. There is a debate in the literature about the effect of age on treatment satisfaction in patients with type 2 diabetes. In the study by Biderman et al. [29], no correlation was found between age and treatment satisfaction, while in the study by Brod et al. [30], such a relationship has been confirmed. On the other hand, in the study by Suzuki et al. [31], younger age had a negative effect on treatment satisfaction. Differences in the authors’ results may be due to differences in the selection of study groups. It is also important to keep in mind that many factors influence the treatment satisfaction assessment. Elderly patients often develop their own beliefs about medications based on their own or their friends’ experiences, and their level of treatment satisfaction may be due to misconceptions or lack of knowledge about the duration of drug action or possible side effects. In addition, older patients with type 2 diabetes are particularly at risk of hypoglycaemia and may be less satisfied with treatments that increase this risk.

This study had several limitations. One of them is that reliability was not assessed using the test–retest method, and another is that CEA was conducted on a relatively small sample of 250 patients [32]. Additionally, an indirect measurement of adherence via self-reported questionnaire, which is prone to underestimation due to patients’ memory problems and desire to conform to society, is another limitation of the study. Nonetheless, self-declaration-based adherence measurement is easy to implement and has been shown to be more reliable than completion history or electronic monitoring. Another limitation of the study is the lack of data on drug interactions. Further studies involving other diseases and drugs are needed to confirm the results. As the different illness groups might by themselves differ in many aspects, this might limit the generalization of results to this and other groups. Furthermore, because of the cross-sectional study design, it was not possible to examine the random effects of low treatment satisfaction on clinically relevant results.

## 5. Conclusions

The SATMED-Q is a good tool for assessing the treatment satisfaction of Polish-speaking patients with chronic diseases and can be recommended for use in everyday clinical practice.

Factors decreasing treatment satisfaction generally are low level of adherence and primary education; for patients with heart failure, the primary factor is living alone; for patients with type 2 diabetes, the primary factor is older age.

## Figures and Tables

**Table 1:** The socio–clinical characteristics of the research group.

Parameter		Group				*p*
		Hypertension (*n* = 55)	Type 2 Diabetes (*n* = 42)	Heart Failure (*n* = 100)	All Patients (*n* = 197)	
Age (years)	mean (SD)	62.51 (14.62)	60.83 (13.23)	67.59 (11.93)	64.73 (13.27)	*p* = 0.003 *
						HF > HTN, DM
Sex	Women	29 (52.73%)	29 (69.05%)	32 (32.00%)	90 (45.69%)	*p* < 0.001 *
	Men	26 (47.27%)	13 (30.95%)	68 (68.00%)	107 (54.31%)	
Level of education	Primary	6 (10.91%)	2 (4.76%)	1 (1.00%)	9 (4.57%)	*p* = 0.012 *
	Vocational	16 (29.09%)	17 (40.48%)	42 (42.00%)	75 (38.07%)	
	Secondary	14 (25.45%)	13 (30.95%)	40 (40.00%)	67 (34.01%)	
	Higher	19 (34.55%)	10 (23.81%)	17 (17.00%)	46 (23.35%)	
Living	With a spouse/partner	30 (54.55%)	22 (52.38%)	51 (51.00%)	103 (52.28%)	*p* = 0.961
	With children	15 (27.27%)	10 (23.81%)	27 (27.00%)	52 (26.40%)	
	Alone	10 (18.18%)	10 (23.81%)	22 (22.00%)	42 (21.32%)	
Disease duration (years)	Up to 5 years	35 (63.64%)	19 (45.24%)	67 (67.00%)	121 (61.42%)	*p* = 0.125
	6–10 years	11 (20.00%)	11 (26.19%)	13 (13.00%)	35 (17.77%)	
	Over 10 years	9 (16.36%)	12 (28.57%)	20 (20.00%)	41 (20.81%)	
Comorbidities	0 or 1	42 (76.36%)	22 (52.38%)	45 (45.00%)	109 (55.33%)	*p* = 0.001 *
	2 or more	13 (23.64%)	20 (47.62%)	55 (55.00%)	88 (44.67%)	
Number of tablets taken	Up to 5	26 (47.27%)	12 (28.57%)	8 (8.00%)	46 (23.35%)	*p* < 0.001 *
	5 and more	28 (50.91%)	23 (54.76%)	71 (71.00%)	122 (61.93%)	
	Does not know	1 (1.82%)	7 (16.67%)	21 (21.00%)	29 (14.72%)	
Hospitalisations	Yes	8 (14.55%)	17 (40.48%)	61 (61.00%)	86 (43.65%)	*p* < 0.001 *
	No	47 (85.45%)	25 (59.52%)	39 (39.00%)	111 (56.35%)	
Number of hospitalisations	0 or 1	54 (98.18%)	32 (76.19%)	64 (64.00%)	150 (76.14%)	*p* < 0.001 *
	2 or more	1 (1.82%)	10 (23.81%)	36 (36.00%)	47 (23.86%)	
ARMS (total score)	Mean (SD)	17.56 (5.21)	18.21 (5.34)	17.23 (4.36)	17.23 (4.85)	*p* = 0.791

Abbreviations: HF—heart failure; DM—diabetes mellitus; HTN—hypertension. *p*—for quantitative variables Kruskal–Wallis test + post hoc analysis (Dunn’s test), for qualitative variables chi-square test or Fisher’s exact test. * Statistically significant difference (*p* < 0.05).

**Table 2:** SATMED-Q results.

SATMED-Q			Group			*p*
		All Patients (*n* = 197)	Hypertension (*n* = 55)	Type 2 Diabetes (*n* = 42)	Heart Failure (*n* = 100)	
SATMED-Q total score	mean (SD)	73.63 (18.42)	76.1 (22.38)	73.81 (20.18)	72.21 (14.98)	*p* = 0.04 *
						HTN > HF
No side effects	mean (SD)	85.32 (28.62)	84.39 (29.14)	74.6 (33.08)	90.33 (25.12)	*p* = 0.008 *
						HF > DM
Effectiveness of treatment	mean (SD)	67.72 (26.47)	70.76 (32.94)	74.8 (28.0)	63.08 (20.56)	*p* = 0.002 *
						HTN, DM > HF
Convenience of use	mean (SD)	76.31 (21.61)	81.06 (25.18)	77.18 (24.0)	73.33 (17.85)	*p* = 0.003 *
						HTN > HF
Impact on daily activities	mean (SD)	66.54 (26.90)	72.27 (30.26)	69.44 (25.08)	62.17 (25.14)	*p* = 0.011 *
						HTN > HF
Medical care	mean (SD)	61.61 (28.36)	63.64 (28.19)	60.42 (24.83)	61.00 (30.01)	*p* = 0.768
					
General satisfaction	mean (SD)	80.29 (22.13)	80.3 (26.85)	81.94 (22.23)	79.58 (19.22)	*p* = 0.206
					

Abbreviations: HF—heart failure; DM—diabetes mellitus; HTN—hypertension. *p*—Kruskal–Wallis test + post hoc analysis (Dunn’s test). * Statistically significant relationship (*p* < 0.05).

**Table 3:** The loadings and Cronbach’s alpha value for individual items.

Subscale	Item	Loading	*p*	Cronbach’s Alpha
No side effects	1	0.986	*p* < 0.001	0.985
	2	0.978	*p* < 0.001	
	3	0.971	*p* < 0.001	
Effectiveness of treatment	4	0.833	*p* < 0.001	0.864
	5	0.838	*p* < 0.001	
	6	0.812	*p* < 0.001	
Convenience of use	7	0.851	*p* < 0.001	0.917
	8	0.859	*p* < 0.001	
	9	0.937	*p* < 0.001	
Impact on daily activities	10	0.854	*p* < 0.001	0.886
	11	0.760	*p* < 0.001	
	12	0.927	*p* < 0.001	
Medical care	13	0.832	*p* < 0.001	0.899
	14	0.982	*p* < 0.001	
General satisfaction	15	0.866	*p* < 0.001	0.885
	16	0.893	*p* < 0.001	
	17	0.793	*p* < 0.001	

**Table 4:** Results of the confirmatory factor analysis.

Chi-Squared Test			RMSEA	CFI	TLI	SRMR
χ^2^	df	*p*				
84.569	113	0.979	<0.001	>0.999	>0.999	0.067

**Table 5:** Results of multiple factor regression analysis.

Factor		Hypertension	Type 2 Diabetes Mellitus	Heart Failure	All Patients
		Parameter	Parameter	Parameter	Parameter
Age	[years]	0.067	0.633 *	0.067	0.099
Sex	Men	−2.706	2.152	−1.712	−1.713
Education	Vocational	8.527	20.05	−11.652	14.576 *
	Secondary	13.059	26.904	−13.915	14.055 *
	Higher	17.334	28.464	−8.114	19.372 *
Living	With children	−5.367	3.156	−3.317	−3.138
	Alone	−1.168	−2.015	−8.71 *	−4.797
Disease duration (years)	6–10 years	0.431	0.205	5.016	5.093
	Over 10 years	−8.073	−5.912	2.553	−2.724
Comorbidities	2 or more	0.631	−8.6	−3.338	−4.159
Number of tablets taken	5 and more	−0.934	0.556	0.038	0.586
	Does not know	−7.301	−4.795	−4.87	−3.993
Hospitalisations due to HF	No	−7.927	−1.251	2.296	1.867
Number of hospitalizations due to HF	2 or more	−1.498	−1.755	1.168	1.684
ARMS	Total score	−2.088 *	−1.879 *	−0.942 *	−1.384 *

*p*—multiple factor linear regression. * Statistically significant relationship (*p* < 0.05). Reference categories: women, primary education, living with a spouse/partner, disease duration up to 5 years, number of tablets taken up to 5, HF hospitalizations, 0 or 1 HF hospitalizations.

## Data Availability

The data presented in this study are available on request from the corresponding author.
